# Private health care market shaping and changes in inequities in childhood diarrhoea treatment coverage: evidence from the analysis of baseline and endline surveys of an ORS and zinc scale-up program in Nigeria

**DOI:** 10.1186/s12939-021-01425-2

**Published:** 2021-03-31

**Authors:** Tiwadayo Braimoh, Isaac Danat, Mohammed Abubakar, Obinna Ajeroh, Melinda Stanley, Owens Wiwa, Marta Rose Prescott, Felix Lam

**Affiliations:** 1 Clinton Health Access Initiative, No. 62, KG5 Avenue, Kacyiru, Kigali, Rwanda; 2 Faculty of Education, Health and Wellbeing, University of Wolverhampton, Wolverhampton, UK; 3 Clinton Health Access Initiative, Abuja, Nigeria; 4 Malaria Consortium, Abuja, Nigeria; 5 Clinton Health Access Initiative, Boston, MA USA

**Keywords:** Inequities, ORS, Zinc, Treatment coverage, Private sector, Socioeconomic status, Location

## Abstract

**Background:**

Nearly 90,000 under-five children die from diarrhoea annually in Nigeria. Over 90% of the deaths can be prevented with oral rehydration salt (ORS) and zinc treatment but coverage nationally was less than 34% for ORS and 3% for zinc with wide inequities. A program was implemented in eight states to address critical barriers to the optimal functioning of the health care market to deliver these treatments. In this study, we examine changes in the inequities of coverage of ORS and zinc over the intervention period.

**Methods:**

Baseline and endline household surveys were used to measure ORS and zinc coverage and household assets. Principal component analysis was used to construct wealth quintiles. We used multi-level logistic regression models to estimate predictive coverage of ORS and zinc by wealth and urbanicity at each survey period. Simple measures of disparity and concentration indices and curves were used to evaluate changes in ORS and zinc coverage inequities.

**Results:**

At baseline, 28% (95% CI: 22–35%) of children with diarrhoea from the poorest wealth quintile received ORS compared to 50% (95% CI: 52–58%) from the richest. This inequality reduced at endline as ORS coverage increased by 21%-points (*P* <  0.001) for the poorest and 17%-points (*P* <  0.001) for the richest. Zinc coverage increased significantly for both quintiles at endline from an equally low baseline coverage level. Consistent with the findings of the pairwise comparison of the poorest and the richest, the summary measure of disparity across all wealth quintiles showed a narrowing of inequities from baseline to endline. Concentration curves shifted towards equality for both treatments, concentration indices declined from 0.1012 to 0.0480 for ORS and from 0.2640 to 0.0567 for zinc. Disparities in ORS and zinc coverage between rural and urban at both time points was insignificant except that the use of zinc in the rural at endline was significantly higher at 38% (95%CI: 35–41%) compared to 29% (95%CI, 25–33%) in the urban.

**Conclusion:**

The results show a pro-rural improvement in coverage and a reduction in coverage inequities across wealth quintiles from baseline to endline. This gives an indication that initiatives focused on shaping healthcare market systems may be effective in reducing health coverage gaps without detracting from equity as a health policy objective.

**Supplementary Information:**

The online version contains supplementary material available at 10.1186/s12939-021-01425-2.

## Introduction

### Background

Oral rehydration solution (ORS) and zinc are effective treatments for diarrhoea in children under-five and universal coverage of these treatments could prevent up to 90% of diarrhoea mortality [1, 2]. Over the last several decades, the global health community has focused efforts to increase use of ORS and zinc in high-burden countries [3]. While many countries have made substantial gains in ORS and zinc coverage, there still remain large in-country variations in coverage associated with various factors [4–8]. The existence of inequities along the dimensions of socioeconomic status (SES) and place of residence have been identified as important reasons for the slow progress towards reducing child mortality in many Low- and Middle-Income countries (LMIC) [9–12]. Disparities in coverage exacerbates health inequities already faced by marginalized populations. For example, children living in poverty already face higher burdens of diarrhoea infection and death due to high prevalence of risk factors such as inadequate access to clean water and sanitation and higher rates of malnutrition [13].

In Nigeria, diarrhoea is a leading cause of death among children younger than 5 years, accounting for nearly 90,000 under-five deaths in 2012 [14]. To address this, the Federal Ministry of Health (FMOH) and the National Primary Health Care Development Agency of Nigeria (NPHCDA) developed the Essential Childhood Medicines Scale-up Plan in 2012 to increase use of ORS and zinc [15]. At the time, less than 34% of children with diarrhoea were receiving ORS and 3% were receiving zinc. Furthermore, despite being at greater risk of dying from diarrhoea, children from poor households were less likely to receive ORS and zinc than children from wealthier households. Analysis from Nigeria’s 2013 Demographic and Health Survey (DHS) found that children with diarrhoea from the richest households had 165% higher treatment rates with ORS than those from the poorest households, and nearly 60% more children living in urban areas were treated with ORS as compared to rural children [16]).

Between 2013 to 2017, the Clinton Health Access Initiative (CHAI), with funding from the Norwegian Agency for Development Cooperation and Global Affairs Canada, supported the Government of Nigeria to implement the Essential Childhood Medicines Scale-up Plan with a focus on eight states - Bauchi, Cross River, Lagos, Kaduna, Kano, Katsina, Niger, and Rivers. These eights states were selected for the program due to their high burden of diarrhoea in addition to being strategic distribution centres for pharmaceutical products. A previous program evaluation found that ORS coverage in the 8 states had increased from 38 to 55% and combined ORS and zinc coverage increased from 4 to 30% over the course of the program [17]. However, the evaluation did not examine whether the program helped reduce inequities in coverage, and it has been argued that interventions oriented towards the private sector may worsen inequities due to the profit motive of private actors [18, 19].

### Study objectives

The objective of the study is to examine how inequities in ORS and zinc coverage may have changed over the course of the program. We hypothesize that inequities in ORS and zinc coverage, as evaluated by SES and urbanicity, decreased over the course of the program.

## Methods

### Program description

The program focused on addressing both key supply and demand challenges that hamper availability, affordability and uptake of ORS and zinc products for childhood diarrhoea treatment. On the supply side, the program collected and analysed market data to generate and share insights such as potential market size, competitive landscape, and return on investment for ORS and zinc with manufacturers and importers in order to stimulate and guide their investment decisions regarding production, promotion, and sale of ORS and zinc. The program also shared the latest evidence on low-osmolarity ORS and zinc with relevant agencies in order to update regulatory guidelines and facilitate product registration and market entry of the optimal products. Furthermore, suppliers were provided with technical assistance on cost reduction, for example through cost-of-goods-sold (COGS) analysis to identify lower cost inputs and packaging optimization. To improve rural availability, suppliers were incentivised through time-limited financial and technical support to develop distribution models that would sustainably reach underserved areas. For example, the program signed agreements with suppliers to share a proportion of the costs for establishing a rural sales force if suppliers met targets on volume and rural availability of ORS and zinc. They were also supported with the development and implementation of innovative marketing and sales strategies. To engender competition and ensure a vibrant market, multiple suppliers were concurrently supported to enter the market. The number of low-osmolarity ORS products competing for market share increased to 35 from just one, and zinc DT increased to 12 and co-packs increased to 10 from zero during the program.

To increase demand, the program conducted trainings and mentoring sessions with health care professionals and patent and proprietary medicines vendors (PPMVs). The trainings were conducted once but three to four cycles of one-on-one mentoring sessions were conducted through follow-up visits to individual providers by specially trained peers to reinforce knowledge and entrench the right practices. PPMVs are largely informal private medicine retailers but they are a dominant source of treatment for childhood illnesses, especially in underserved communities [20]. Also, key influencers and various community networks were engaged to reach mothers and caregivers with messages on the right steps to take for children with diarrhoea.

### Study design

The study design used baseline and endline household surveys to measure changes in the coverage of ORS and zinc for the treatment of diarrhoea in children under-five. In addition, the surveys also collected data on household assets in order to measure household wealth. The household surveys were modelled using standardized questions from the DHS and Multiple Indicator Cluster Survey (MICS). Further details on the study design have been published elsewhere [17]. The study was reported according to the Strengthening the Reporting of Observational Studies in Epidemiology Statement [21].

### Setting

The study was conducted in the eight Nigerian program states. Nigeria is the most populous country in Africa with population estimated at nearly 200 million [22]. Children less than 5 years account for 17.6% of the population and 51% are from rural areas. There are wide wealth disparities across the country with the northern part of the country being the poorest. On the average, 54% of the population live below international poverty line of US$1.25 per day [23]. Nigeria operates a mixed system with a three-tiered public health system co-existing with a heterogenous private sector. The private sector provides about 60% of health service delivery [24]. Nearly two-thirds of children are brought to PPMVs for treatment. The eights states where the program was implemented account for 40% of the burden of childhood diarrhoea [25]. Three of the states are in the South and five are in the North of the country.

### Study population

The study population were children under five who resided in one of the eight states within Nigeria where the program was implemented and had diarrhoea within the past 2 weeks preceding the survey. The study used a stratified, multi-stage cluster randomized sampling design for identifying and selecting study participants which is described elsewhere [17].

### Data sources

Data were collected using population-based household surveys among caregivers of children under five. The baseline survey was conducted between December 2013 and November 2014 and the endline survey was conducted between April 2016 and May 2017. The surveys collected information on household characteristics including asset ownership, caregiver knowledge, care seeking behaviour, geographical location, caregiver and child sex and age, prevalence of diarrhoea, and treatment practices.

### Variables

The primary study outcome was treatment of diarrhoea with ORS and zinc by children who had diarrhoea within the last two weeks of the survey. The survey used standardized questions adopted from the DHS and MICS to measure ORS and zinc coverage. The primary caregiver of children in each household were asked whether any child had diarrhoea in the last 2 weeks preceding the survey. Diarrhoea was defined as passing three or more loose stools in a 24 h period. For children who did have diarrhoea within the last 2 weeks preceding the survey, caregivers were asked if anything was used to treat the diarrhoea, including a fluid made from a packet called ORS, zinc tablets, or zinc syrup.

The survey also collected information on household assets, such as ownership of land, vehicles, farm animals, household goods, and structure of living accommodations. The complete list of household asset questions is presented in the supplementary appendix S1. These household asset questions were also adopted from model DHS and MICS surveys.

Households were designated as living in urban or rural areas based on their census enumeration block. We obtained census enumeration maps from Nigeria’s Bureau of Statistics (NBOS) and National Population Commission (NPC). Each of the sampled census enumeration areas were pre-assigned as urban or rural by NBOS and NPC.

### Sample size

The sample size calculation for the study was designed to detect change in ORS and zinc coverage between the baseline and endline surveys. We used the following formula to calculate the sample size for the study:

$$ N= Deft\ast \frac{{\left({z}_{a/2}-{z}_b\right)}^2\left[{p}_1\left(1-{p}_1\right)+{p}_2\left(1-{p}_2\right)\right]}{{\left({p}_1-{p}_2\right)}^2} $$

where N is the desired sample sizes of children with diarrhoea in each state (assuming one child per household), Deft is the design effect due to clustering which we assumed to be 1.5 [16], p1 is the 2011 state coverage estimates (the most recent coverage data available at the time) [26], and p2 is the endline estimates necessary to see a 25% difference over time [27]. We used a two-sided t test with 95% confidence, 80% power, and equal variances. We took into account the prevalence of diarrhoea found and allowed for a 5% non-response rate [26]. To simplify training and field work management, assuring better data quality, we used the largest sample size required (ie, Lagos) as the sample size for all states. In each state, the sample size was 940 households with a child under five. Based on the lower density of population within Cross Rivers and Rivers, the sample size was reduced to 930 households with children under five.

### Statistical analysis

Stata version 14 (Stata Corp, College Station TX, USA) software was used in the first step of our analyses. We constructed wealth quintiles (poorest, second, middle, fourth, and richest) using principal component analysis of the household assets. Wealth quintiles were used as a measure of SES. Treatment coverage was defined as the percentage of children with diarrhoea in the 2 weeks preceding survey who were treated with ORS and zinc. We conducted descriptive statistics for characteristics of the child, caregiver, and household for each survey period. Standard errors and 95% confidence intervals were estimated using Taylor linearized methods. Pearson’s chi-squared tests were used to compare characteristics and outcomes between baseline and endline surveys and estimate *p*-values. To account for potential confounding due to differences in population characteristics between baseline and endline and adjust for sampling design, we conducted multi-level, mixed-effects logistic regression modelling with ORS, zinc, and combined ORS and zinc use as the dependent outcome and survey period and population characteristics as independent predictors. Using the model results, we constructed predictive probabilities of ORS, zinc, and combined ORS and zinc coverage at baseline and endline and for all sub-populations at both survey periods. Analyses were stratified by geographical location and SES. All analyses incorporated sampling weights and took into account clustering at the enumeration area and household levels.

Microsoft Excel was used to compute simple absolute and relative disparities for pairwise comparisons and summary measures of inequality [28, 29]. As SES is comprised of ordered subgroups, a single comparison between the richest quintile and the poorest quintile was done, with the former being the reference group (28). Absolute disparities (AD) and relative disparities (RD) were determined as follows:

*AD = y2 – y1*, where *y2* represent coverage in the reference group and *y1* refers to coverage in the comparison group. *RD = y2/y1*, where y2 represent coverage in the reference group and y1 represent coverage in the comparison group.

Concentration curves and indices were employed where the objective was to provide a summary measure across multiple subgroups, which was the case with SES [30]. Concentration curves provide a visual representation of inequality. Concentration curves above the hypothetical line of equality (45–degree line) implies that coverage is concentrated among the poor, below the line of equality mean that coverage is concentrated among the rich and along the line of equality implies equality between groups [29]. Concentration curve was computed with the cumulative percentage of children treated with each of ORS and zinc plotted on y-axis and the cumulative percentage of the population of children with diarrhoea ranked by SES, beginning with the poorest and ending with the richest plotted on the x-axis.

According to Wagstaff et al. (1991), the concentration index is the most appropriate measure of health inequality since it reflects the experiences of the entire population and is sensitive to changes in the distribution of the population across subgroups [31]. As against Erreygers concentration index which has been proposed for ordinal health indicators such as self-reported health [32], Wagstaff indices were used here since the indicator of interest (treatment of childhood diarrhoea with ORS and zinc) were not ordinal and required no scaling.

Defined as twice the area between the concentration curve and the line of equality, the concentration index (C.Index) was computed using the following formula (Fuller and Lury, 1977):

$$ \mathrm{C}.\mathrm{Index}=\left({\mathrm{P}}_1{\mathrm{L}}_2-{\mathrm{P}}_2{\mathrm{L}}_1\right)+\left({\mathrm{P}}_2{\mathrm{L}}_3-\mathrm{P}3{\mathrm{L}}_2\right)+\dots +\left({\mathrm{P}}_{\mathrm{T}-1}{\mathrm{L}}_{\mathrm{T}}-{\mathrm{P}}_{\mathrm{T}}{\mathrm{L}}_{\mathrm{T}-1}\right) $$

where Pt is the cumulative percentage of the sample ranked by economic status in group t, and Lt is the corresponding concentration curve ordinate. T is the number of socio-economic groups or wealth quintiles [29].

Concentration Index takes on values between − 1 and + 1 with 0 representing equality. The index quantifies the degree of relative inequality among subgroups and indicate the extent to which coverage is concentrated among the advantaged or disadvantaged. The larger the absolute value, the greater the disparity. A positive index is obtained when the curve lies below the diagonal (C.Index > 0) indicating that coverage is higher among the richer groups while a negative index is obtained when the curve lies above the diagonal (C.Index < 0) indicating that coverage is higher among the poor [29].

## Results

### Study sample characteristics

The characteristics of surveyed households, caregivers and children have been described elsewhere [17]. But for a few exceptions, there was no statistically significant difference in sample characteristics between endline and baseline (Table 1). At endline, 22% (95% confidence interval (CI) = 20–24%) of diarrhoea episodes were among children 0–11 month compared to 12% (95% CI = 10–15%) at baseline. At endline, 94% (95% CI = 92–96%) of the caregiver respondents were female compared to 90% (95% CI = 88–92%) at baseline. At endline, 55% (95% CI = 51–59%) of diarrhoea episodes came from rural areas whereas at baseline the figure was 65% (95% CI = 60–69%). It was also found that a lower proportion of diarrhoea episodes came from Rivers state (*P* = 0.05) and Bauchi state (*P* = 0.03) at endline as compared to baseline.

**Table 1:** Distribution of demographic and socioeconomic Characteristics by surve

Charactristics	Baseline(***n*** = 1661) %, 95 confidence interval	Endline(***n*** = 2268)%, 95 confidence interval	Endline vs. Baseline ***P***-value*
**Sex of Child**			
Female	48 (45–52)	47 (45–49)	0.769
**Age of Child (months)**			
0–11	12 (10–15)	22 (20–24)	< 0.001
12–23	30 (27–32)	32 (30–34)	0.170
24–35	19 (17–22)	23 (21–25)	0.083
36–47	19 (17–22)	15 (13–17)	0.005
48–59	20 (17–22)	9 (8–10)	< 0.001
**Source of care**			
Did not seek care or advice outside the home	34 (30–38)	27 (25–30)	0.005
Source care in public sector	25 (22–28)	27 (24–30)	0.436
Source care in private sector	34 (30–38)	38 (35–41)	0.093
Source care in other place	2 (1–3)	3 (2–4)	0.200
Source care from multiple sectors	5 (4–7)	5 (4–7)	0.901
**Sex of respondent/child caregiver**			
Female	90 (88–92)	94 (92–96)	0.013
**Age (years) of respondent/child’s caregiver**			
15–19	4 (3–6)	3 (2–4)	0.027
20–29	49 (45–53)	53 (50–56)	0.122
30–39	31 (27–34)	33 (30–36)	0.325
40–49	11 (8–15)	8 (6–9)	0.035
50–59	3 (2–5)	2 (2–4)	0.482
60+	2 (1–3)	1 (1–2)	0.310
**Child’s caregiver attended any level of schooling**	51 (46–55)	56 (52–61)	0.056
**Residence of Household**			
Rural	65 (60–69)	55 (51–59)	0.002
**Size of household**			
2–4	29 (26–33)	32 (29–35)	0.548
5–7	39 (35–43)	40 (38–43)	0.104
8–10	18 (16–21)	16 (13–18)	0.420
11+	13 (11–16)	12 (10–15)	0.384
**State where program was implemented**			
Lagos	8 (5–12)	11 (9–13)	0.268
Kano	21 (17–25)	23 (20–27)	0.309
Rivers	10 (8–13)	6 (4–8)	0.005
Bauchi	14 (12–18)	10 (7–13)	0.030
Cross River	7 (5–9)	6 (5–7)	0.794
Kaduna	14 (10–18)	16 (13–19)	0.399
Katsina	14 (12–17)	16 (13–18)	0.454
Niger	12 (10–15)	13 (11–15)	0.495
**Wealth quintiles of households**			
Poorest	20 (17–24)	22 (19–26)	0.446
Second	19 (16–23)	21 (19–24)	0.422
Middle	20 (16–24)	22 (19–24)	0.538
Fourth	19 (16–23)	18 (16–22)	0.723
Richest	21 (17–26)	17 (14–20)	0.092

*P-values were generated using Pearson’s chi-squared tests

### ORS and zinc treatment coverage among children with diarrhoea by subgroups

Table 2 presents coverage by geographical location and SES. From baseline to endline. ORS coverage increased by 18 percentage points (*P* <  0.001) in rural but improvements for urban (6 percentage points) was not statistically significant (*P* = 0.210). Conversely, zinc coverage increased significantly for both rural and urban from baseline to endline. ORS coverage increased by 21 percentage points (*P* <  0.001) for the poorest and 17 percentage points (*P* < 0.001) for the richest. Zinc coverage increased significantly for both quintiles at endline from an equally low baseline coverage level. Across wealth quintiles, ORS coverage increased significantly from baseline to endline except for the middle quintile with 4 percentage points increase (*P* = 0.462) and fourth quintile with 7 percentage points (*P*-value = 0.242). For zinc, coverage increased significantly for all quintiles. Coverage results for the combined use of both ORS and zinc is presented in supplementary appendix S2.

**Table 2:** ORS and zinc treatment coverage by subgroup

	Received ORS			Received Zinc		
	Baseline ***n*** = 1349%, 95 confidence interval	Endline ***n*** = 2023%, 95 confidence interval	Endline - Baseline Difference 95% confidence interval, ***P***-value*	Baseline ***n*** = 1333%, 95 confidence interval	Endline ***n*** = 2000%, 95 confidence interval	Endline - Baseline Difference 95% confidence interval, ***P***-value*
**Residence of households**						
Urban	44 (36–51)	50 (49–54)	6 (− 3.2–15), 0.210	6 (3–9)	29 (25–33)	23 (18–28), < 0.001
Rural	39 (35–42)	57 (53–60)	18 (13–23), < 0.001	5 (4–7)	38 (35–41)	33 (29–36), < 0.001
**Wealth quintiles**						
Poorest	28 (22–35)	50 (44–56)	21 (12–31), < 0.001	3 (1–5)	35 (30–40)	33 (27–38), < 0.001
Second	37 (28–46)	51 (46–57)	15 (4–25), 0.006	3 (1–6)	31 (25–36)	27 (21–33), < 0.001
Middle	43 (34–52)	47 (41–53)	4 (−7–15), 0.462	4 (1–6)	25 (20–29)	21 (16–26), < 0.001
Fourth	47 (37–56)	54 (47–60)	7 (−5–18), 0.242	7 (3–10)	33 (29–38)	27 (21–32,), < 0.001
Richest	50 (52–58)	67 (61–73)	17 (7.3–27), 0.001	10 (5–15)	40 (40–52)	36 (28–44), < 0.001

*P-values were generated using Wald’s chi-squared tests

### Inequalities in ORS and zinc treatment coverage

#### Geographical location

For both ORS and zinc, absolute and relative disparity measures show pro-urban skewness at baseline, but this was reversed in favour of rural at endline (Table 3). However, the disparities in both time points were not statistically significant except for zinc which had a coverage of 38% (95% CI = 35–41%) in the rural and 29% (95% CI = 25–33%) in the urban at endline (Tables 2).

**Table 3:** Disparities in ORS and zinc treatment coverage for children with diarrhoea in urban versus rural households

	ORS				zinc			
Time	Treatment Coverage %		Difference % points (Urban - Rural)	Ratio (Urban/Rural)	Treatment Coverage %		Difference% points (Urban - Rural)	Ratio (Urban/Rural)
	Urban	Rural			Urban	Rural		
Baseline	43.81	38.51	5.30	1.14	5.58	5.16	0.42	1.08
Endline	49.55	56.78	−7.23	0.87	28.59	37.80	−9.21	0.76

#### Socioeconomic status

There were wide disparities in ORS coverage between wealth quintiles at baseline. The poorest had a significantly lower coverage of 28% (95% CI = 22–35%) as compared to the fourth quintile with 47% (95% CI = 37–56%) and richest with 50% (95% CI = 52–58%). However, the magnitude of coverage increase from baseline to endline was higher for the poorest, resulting in the narrowing of disparity gaps at endline (Table 2). Pairwise comparisons of the richest and the poorest show a decline in absolute disparity for ORS from 21 percentage points at baseline to 17 percentage points at endline. In relative terms, the richest had 76% higher coverage at baseline but at endline, the advantage over the poorest declined to 34% (Table 4). In the case of zinc, absolute and relative disparities went different directions. Absolute disparities for zinc was higher (11%) at endline relative to baseline (7%), both in favour of the richest. In contrast, the relative disparity for zinc decreased from a richest/poorest ratio of 3.5 to 1 at baseline to a ratio of 1.3 to 1 at endline (Table 4).

**Table 4:** absolute and relative disparities between the richest and the poorest socioeconomic groups

	`	Treatment Coverage					Difference % points	Ratio
		Poorest %	Second %	Middle %	Fourth %	Richest %		
**ORS**	**Baseline**	28.38	36.63	42.86	46.8	49.82	21.44	1.76
	**Endline**	49.87	51.39	46.89	53.5	67.03	17.16	1.34
**zinc**	**Baseline**	2.81	3.4	3.6	6.7	9.89	7.08	3.52
	**Endline**	35.47	30.6	24.57	33.32	46.01	10.54	1.30

**Fig. 1:**
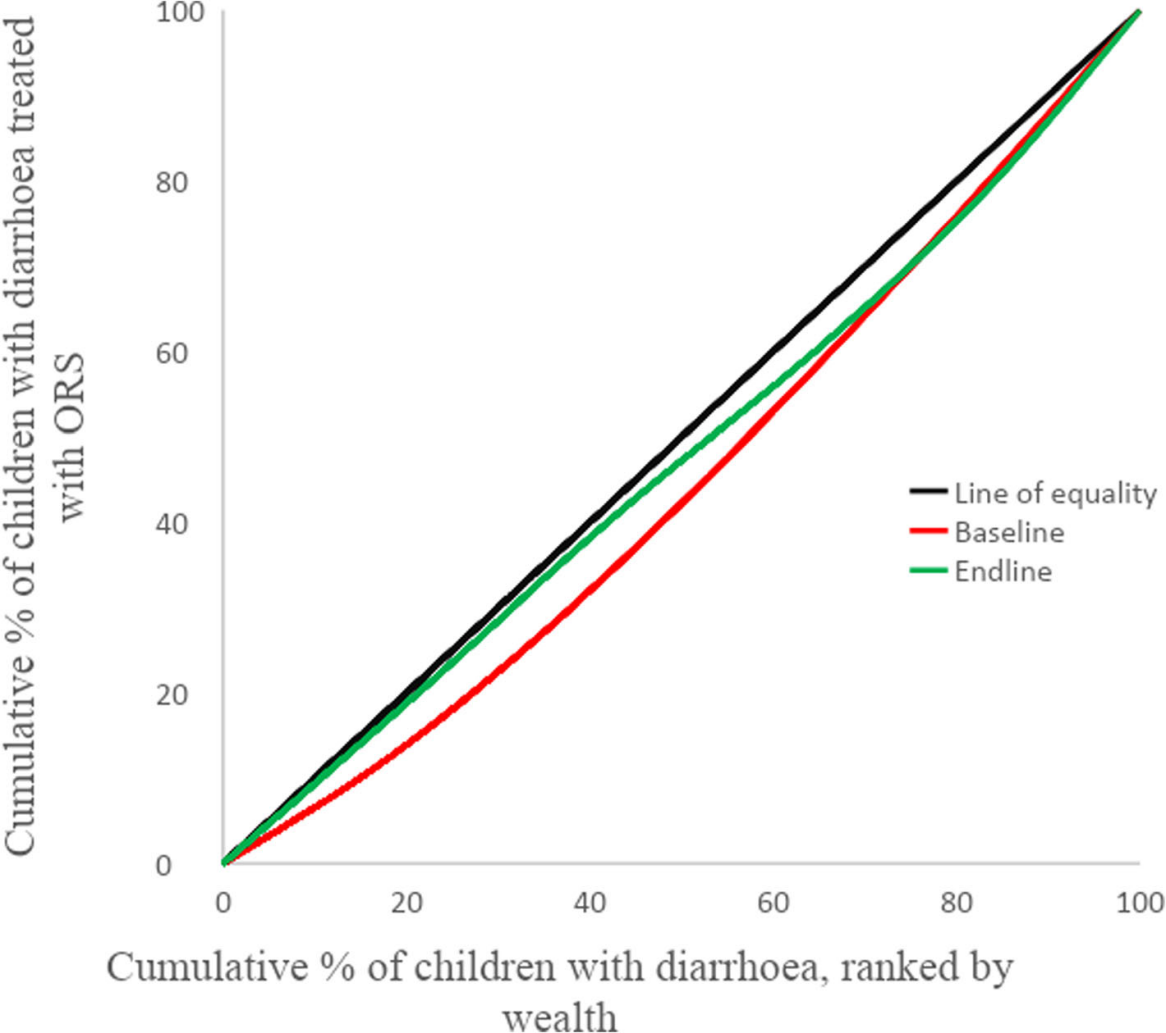
Concentration curve for ORS treatment coverage among socioeconomic groups

Figure 1 shows concentration curve for ORS coverage. At baseline, the curve lies below the 45–degree line (line of equality) implying that coverage was concentrated among the rich. At endline the curve moved closer to the line of equality, implying that coverage became more equally distributed within the wealth subgroups. The concentration index for ORS at baseline was 0.1012 indicating a pro-rich skew in coverage. At endline, the concentration index was 0.048 indicating that coverage across wealth quintiles became closer to equality, though it shows that coverage is still slightly to the advantage of the rich [33].

**Fig. 2:**
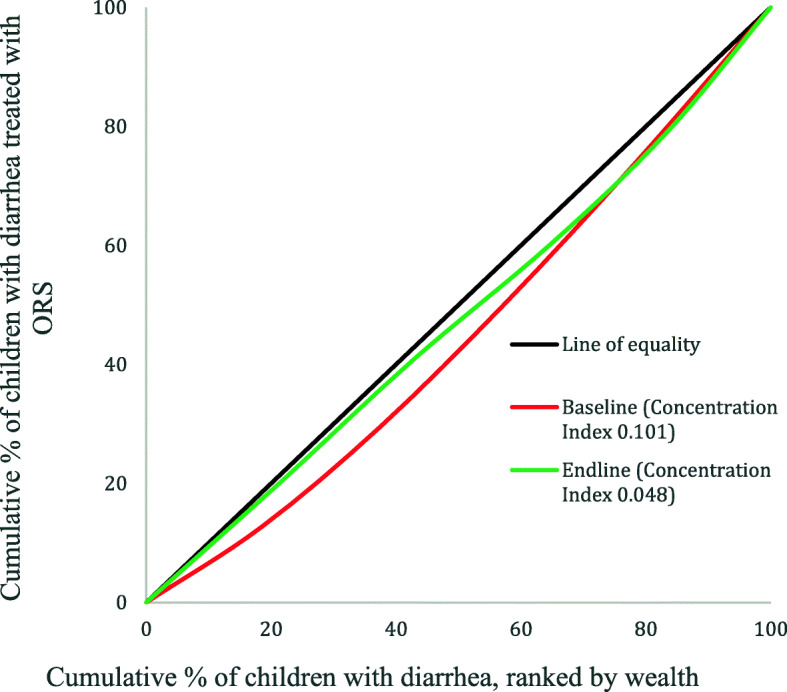
Concentration curve for zinc treatment coverage among socioeconomic groups

Figure 2 shows concentration curve for zinc coverage. The curve at baseline lies farther below the line of equality meaning that coverage was concentrated among the rich. At endline, it moved to roughly overlap the line of equality implying a shift towards coverage equality across wealth quintiles. In congruence with the curves, the concentration index changed from 0.264 at baseline to 0.057 at endline, implying a shift towards equality, but there was still a slight skewness of coverage in favour of the rich. The results of analyses on the inequality of coverage for the combined use of ORS and zinc is presented in supplementary appendix S3.

## Discussion

The results show a pro-rural improvement in coverage and a significant reduction in coverage inequality between wealth quintiles from baseline to endline for both products. For each of ORS and zinc, the analyses demonstrate a considerable deviation from equality at baseline but the results at endline indicate a narrowing of equity gaps over the intervention period. Although zinc had a higher relative inequality at baseline as compared to ORS, the number of children affected by ORS inequality was greater. The seeming conflict in the result of the pairwise comparison of zinc coverage for the poorest and richest was resolved as a closer look at Table 4 reveals poor baseline values for both wealth quintiles. The high relative disparity at baseline only reflects the relative scale of coverage despite the low coverage for both quintiles. This result tends to support arguments for the use of multiple measures to enable more accurate interpretation of equity analysis [34]. As reasoned by O’Donnell et al. (2008), equity is an important health policy goal, but the average level of health should not be overlooked [29]. In health, inequality is the metric by which inequity can be assessed. The former indicate situations of the latter when differences in health between social groups are unjust or unfair [28].

Previous studies in Africa, India and Bangladesh suggest childhood diarrhoea treatment reflect differences in SES (the poor being less likely to afford treatments); geographical location of residence (people in rural and hard-to-reach areas being more likely to have to travel farther and for longer to get treatment); education (the less educated being least likely to know how to treat or seek treatment for a child with diarrhoea); gender (caregivers being less likely to seek treatment for girls than for boys); and ethnicity (where one ethnic group is better positioned than others to access treatments) [9–12, 35]. Our study aimed to examine changes in ORS and zinc coverage inequities by the dimensions of SES and geographical location over the period of a program utilizing a market-based approach for improving treatment of childhood diarrhoea.

Due to the observational design of the study, we are unable to isolate the effect of the program from external and confounding factors that could have affected coverage, such as other child health programs and economic shocks. Demographic characteristics of the sample population also had significant differences between the baseline and endline surveys. This may have been due to higher diarrhoea prevalence found at endline particularly in urban areas than was found at baseline. While we controlled for differences in observable population characteristics, such as ruralness, we cannot rule out that there are unobserved population characteristics associated with ORS and zinc use. In addition, asset-based wealth indices have been criticised for various reasons including the assessment of only relative rather than absolute SES. This may have affected the results, if for instance, the absolute wealth of households in the poorest quintile at baseline is comparable with that of the middle quintile at endline. Nevertheless, wealth indices have been shown to be comparable with other more complex indicators of SES and relevant in developing countries [34, 36].

Despite the limitations, it is evident from the results that inequities reduced from baseline to endline. Although we are not able to draw a causal association to this result, the large magnitude of observed changes particularly with respect to SES and the consistency of the results suggest that the market shaping intervention is probably responsible for the outcomes. The direction of the inequalities obtained from the pairwise comparisons of urban and rural were similar to that of the richest and poorest groups, even though, urban-rural disparities were found to be statistically non-significant except for zinc at endline,. This seems consistent with the report by Doherty et al. (2015) that inequities arising from SES are closely associated with geographical dimension of disparities [37].

As wealth subgroups have a natural ordering, single comparison using absolute and relative measures between the richest and the poorest leaves out the three middle quintiles. This justified the use of concentration curves and indices.

The concentration curves give a visual representation of a reduction in inequality across the wealth quintiles and the indices indicates the magnitude of the decline towards zero. While the cut off for concentration index is often based on social value judgement, an absolute value greater than 0.2 is considered to represent a high level of relative inequality [28]. Examining both the concentration curves and indices in conjunction with the simple measures, one can ensure that no information is lost [29].

This finding corroborates other studies documenting reductions in inequalities in childhood diarrhoea treatment coverage after targeted interventions. Larson et al. (2009) reported on the efforts to deliver zinc at scale in Bangladesh through the “Scaling Up of Zinc for Young Children (SUZY)” project and concluded that there was an increase in zinc coverage over time and a reduction in gaps based on income status [4].

The decline in inequality levels may have been driven by the approach of targeting interventions such as the training and mentoring of PPMVs, the predominant provider for disadvantaged populations [20]. A systematic review published in 2014 reported that interventions that were effective in reducing inequity among different sociodemographic groups included those that use human resources nearest to residents at the community level [38]. Without targeting, health interventions tend to be adopted initially by the wealthiest, and later trickle down to the rest of the population [39–41].

Notwithstanding, there are legitimate concerns about a reliance on the private sector for population health needs. Some argue that they do not have equity as an objective and often locate where willingness to pay is greatest [4, 42]. Studies have shown that urban and wealthier households disproportionately use the private sector compared to rural and poorer households [43, 44]. Conversely, low quality private service provision such as the case of informal providers in rural areas of low-income settings are used disproportionately by the poor [45].

Some proponents of private sector engagement argue for the use of regulation to address these drawbacks but Montagu and Goodman (2016) reasoned that regulation alone can be ineffective in contexts where there is a dearth of credible alternatives to what is being regulated [45, 46]. Moreover, many LMIC country governments lack capacity to provide effective regulation. Market shaping can be effective since it identifies and addresses contextual constraints to harnessing private sector potentials for public health in supportive manner. For example, it was reported that in Tanzania, medicines produced locally were found to be more likely than imported products to be available to rural dwellers [47]. In such situation, market shaping intervention to optimize local manufacture would be an effective approach.

The findings of this study have implications for policy. First, it is important for governments and other actors in global health to know and be proactive to take hold of unutilized contextual levers that have the potential of complementing traditional approaches to deliver high-impact results for those in pressing need. However, while market shaping interventions can be employed in the short run to surmount critical health access challenges, governments should not abdicate their responsibilities of stewarding and ensuring that health programs do not deviate from health policy objectives. Second, although seemingly effective, market shaping programs should not be taken as substitutes to a deliberate effort at building robust and sustainable health systems that meet the health needs of everyone irrespective of their ability to pay. Third, mainstreaming equity considerations into health policies and programmes can help to speed up progress towards universal health coverages. However, undue emphasis on equity to the detriment of overall improvements of outcomes should be avoided. And finally, to target interventions to disadvantaged populations is not the same as addressing the underlying determinants of inequities. Governments must acknowledge and tackle the structural obstacles to equitable access to healthcare to adequately address the unjust differences in health utilization and outcomes.

## Conclusions

This study aimed to contribute to evidence as to whether market shaping interventions in public health programs help to move towards or detracts from equitable treatment coverage for childhood diarrhoea in Nigeria. The results show a pro-rural improvement in coverage and a reduction in coverage inequities across wealth quintiles from baseline to endline. This gives an indication that initiatives focused on shaping healthcare market systems may be effective in reducing health coverage gaps without detracting from equity as a health policy objective. 

## Supplementary Information

**Additional file 1.**

## Data Availability

Data are available from the authors upon reasonable request and with permission of Clinton Health Access Initiative.
