# Supplementary material for: Private health care market shaping and changes in inequities in childhood diarrhoea treatment coverage: evidence from the analysis of baseline and endline surveys of an ORS and zinc scale-up program in Nigeria
Source: Int J Equity Health. 2021 Mar 31;20:88. doi: 10.1186/s12939-021-01425-2 (PMC8011378; doi:10.1186/s12939-021-01425-2)
Supplement: Supplementary file 1 — Additional file 1. [file 12939_2021_1425_MOESM1_ESM.docx]

Supplementary Material

Braimoh et al. Private health care market shaping and changes in inequities in childhood diarrhoea treatment coverage: Evidence from the analysis of baseline and end line surveys of an ORS and zinc scale-up program in Nigeria

Contents

[Appendix S1. Variables used in principal component analysis for wealth index 2](#_Toc63175048)

[Appendix S2. ORS and zinc combined treatment coverage by subgroups 5](#_Toc63175049)

[Appendix S3. Inequalities in combined ORS and zinc treatment coverage 5](#_Toc63175050)

# Appendix S1. Variables used in principal component analysis for wealth index

| **HOUSEHOLD CHARACTERISTICS (HC)** | | | |
| --- | --- | --- | --- |
|  | *Main material of the dwelling floor.*  *Record observation.* | Natural floor (Earth/Sand/Dung) 10  Rudimentary floor (Wood planks/Bamboo) 20  Finished floor (Polished wood/Cement/Carpet/Concrete) 30  Other (*specify*) 96  Don’t know 98 |  |
|  | *Main material of the roof.*  *Record observation.* | Natural roofing (No roof/Thatch/Palm leaf) 10  Rudimentary Roofing (Palm fronds/Bamboo/Wood planks/Plastic/Cardboard/Nylon/Polythene) 20  Finished roofing (Zinc/Metal/Wood/Ceramic tiles/Concrete/Asbestos/Sheets/Shingles) 30  Other (*specify*) 96  Don’t know 98 |  |
|  | *Main material of the exterior walls.*  *Record observation.* | Natural walls (Cane /Palm/Trunks/Mud and sticks/ Straw/Thatch mats) 10  Rudimentary walls (Mud bricks/Plywood/Reused wood/Cardboard and plastic) 20  Finished walls (Cement/Stone blocks/Bricks/Wood planks/Shingles) 30  Other (*specify*) 96  Don’t know 98 |  |
|  | Does your household have:  [A] Electricity?  [B] A radio?  [C] A television?  [D] A mobile telephone?  [E] A landline telephone?  [F] A refrigerator?  [G] Cable TV?  [H] A Generator?  [I] Air conditioner?  [J] A computer?  [K] Electric iron?  [L] A fan? | Yes No Ref DK  A. Electricity 1 2 7 8  B. Radio 1 2 7 8  C. Television 1 2 7 8  D. Mobile phone 1 2 7 8  E. Landline 1 2 7 8  F. Refrigerator 1 2 7 8  G. Cable TV 1 2 7 8  H. Generator 1 2 7 8  I. Air conditioner 1 2 7 8  J. Computer 1 2 7 8  K. Electriciron 1 2 7 8  L. Fan 1 2 7 8 |  |
|  | Does any member of your household own:  [A] A watch?  [B] A bicycle?  [C] A motorcycle or scooter?  [D] An animal-drawn cart?  [E] A car or truck?  [F] A boat with a motor?  [G] A canoe? | Yes No Ref DK  A. Watch 1 2 7 8  B. Bicycle 1 2 7 8  C. Motorcycle/ Scooter 1 2 7 8  D. Animal drawn-cart 1 2 7 8  E. Car / Truck 1 2 7 8  F. Boat with a motor 1 2 7 8  G. Boat without a motor 1 2 7 8 |  |
|  | What is the main source of drinking water for members of your household? | Piped Water 11  Tube well or borehole 12  Dug well 13  Water from spring 14  Rain water 15  Tanker truck 16  Vendor (Mai ruwa) 17  Surface water (river/dam/lake/pond/stream/  canal/irrigation channel) 18  Bottled water / Water dispenser 19  Sachet water (Pure water) 20  Other (specify) 96  Refused 97  Don’t know 98 |  |
|  | What type of fuel does your household mainly use for cooking? | Electricity 11  Gas 12  Kerosene / Paraffin 13  Charcoal 14  Firewood 15  Straw / Shrubs / Grass 16  Agricultural crop (e.g. corn stalk) 17  Animal dung 18  No food cooked in household 95  Other (specify) 96  Refused 97  Don’t know 98 |  |
|  | Does any member of this household own any land that can be used for agriculture? | Yes 1  No 2  Refused 7  Don’t know 8 |  |
|  | Does this household own any livestock, herds, other farm animals, or poultry? | Yes 1  No 2  Refused 7  Don’t know 8 | 1 🡪 HC10  2 🡪 HC11  7 🡪 HC11  8 🡪 HC11 |
|  | How many of the following animals does this household have?  [A] Cattle, milk cows, or bulls?  [B] Horses, donkeys, or mules?  [C] Goats?  [D] Sheep?  [E] Chickens?  [F] Pigs?  Write the number on line  Special codes:  If none, record ‘00’.  If 95 or more, record ‘95’.  If refused, record ‘97’.  If unknown, record ‘98’. | Cattle, milk cows, or bulls ___ ___  Horses, donkeys, or mules ___ ___  Goats ___ ___  Sheep ___ ___  Chickens ___ ___  Pigs ___ ___ |  |

# Appendix S2. ORS and zinc combined treatment coverage by subgroups

|  | **Combined ORS and Zinc** | | |  |
| --- | --- | --- | --- | --- |
|  | **Baseline  n = 1333 %, 95% confidence interval** | **Endline  n = 2000 %, 95% confidence interval** | **Endline - Baseline Difference 95% confidence interval, *P*-value*** |  |
| **Residence of households** |  |  |  |  |
| Urban | 4 (1 - 7) | 24 (21 - 28) | 21 (16 - 25), < 0.001 |  |
| Rural | 4 (3 - 5) | 33 (30 - 36) | 29 (26 - 32), < 0.001 |  |
| **Wealth quintiles** |  |  |  |  |
| Poorest | 1 (0.18 - 0.2) | 30 (25 - 34) | 28 (24 - 33), < 0.001 |  |
| Second | 2 (0.3 - 5) | 26 (22 - 31) | 24 (19 - 29), < 0.001 |  |
| Middle | 2 (0.6 - 4) | 20 (16 - 24) | 18 (13-22), < 0.001 |  |
| Fourth | 3 (1 - 5) | 30 (26 - 35) | 27 (22 - 32,), < 0.001 |  |
| Richest | 10 (5 - 16) | 41 (35 - 47) | 31 (23 - 31), < 0.001 |  |
| **P*-values were generated using Wald’s chi-squared tests | | | |  |

# Appendix S3. Inequalities in combined ORS and zinc treatment coverage

1. Concentration curve for combined ORS and zinc treatment coverage among socioeconomic groups
2. Disparities in combined ORS and zinc treatment coverage for children with diarrhoea in urban versus rural households.

| **Time** | **Treatment Coverage  %** | | **Difference  % points** (Urban - Rural) | **Ratio** (Urban/Rural) |
| --- | --- | --- | --- | --- |
|  | Urban | Rural |  |  |
| **Baseline** | 3.92 | 3.77 | 0.15 | 1.04 |
| **Endline** | 24.42 | 32.78 | -8.36 | 0.74 |

1. Disparities in combined ORS and zinc treatment coverage between the richest and the poorest socioeconomic groups

| ` | **Treatment Coverage** | | | | | **Difference  % points** | **Ratio** |
| --- | --- | --- | --- | --- | --- | --- | --- |
|  | **Poorest %** | **Second %** | **Middle %** | **Fourth %** | **Richest %** |  |  |
| **Baseline** | 1.19 | 2.37 | 2.22 | 3.02 | 10.2 | 9.01 | 8.57 |
| **Endline** | 29.67 | 26.25 | 19.9 | 30.15 | 41.07 | 11.4 | 1.38 |
